# Improved Overall Survival, Relapse-Free-Survival, and Less Graft-vs.-Host-Disease in Patients With High Immune Reconstitution of TCR Gamma Delta Cells 2 Months After Allogeneic Stem Cell Transplantation

**DOI:** 10.3389/fimmu.2019.01997

**Published:** 2019-08-22

**Authors:** Lia Minculescu, Hanne Vibeke Marquart, Lars Peter Ryder, Niels Smedegaard Andersen, Ida Schjoedt, Lone Smidstrup Friis, Brian Thomas Kornblit, Søren Lykke Petersen, Eva Haastrup, Anne Fischer-Nielsen, Joanne Reekie, Henrik Sengelov

**Affiliations:** ^1^Department of Clinical Immunology, Copenhagen University Hospital, Rigshospitalet, Copenhagen, Denmark; ^2^Department of Hematology, Copenhagen University Hospital, Rigshospitalet, Copenhagen, Denmark; ^3^Department of Infectious Diseases, PERSIMUNE, Copenhagen University Hospital, Rigshospitalet, Copenhagen, Denmark

**Keywords:** allogeneic stem cell transplantation, innate immunology, gamma delta (γδ) T cells, transplant immunology, cell therapy

## Abstract

T-cell receptor (TCR) γδ cells are perceived as innate-like effector cells with the possibility of mediating graft-vs. -tumor (GVT) without causing graft-vs.-host disease (GVHD) in the setting of hematopoietic allogeneic stem cell transplantation (HSCT). We conducted a prospective study to assess the clinical impact of TCR γδ cell immune reconstitution on overall survival, relapse-free-survival, relapse and GVHD. The impact of CD3, CD4, and CD8 T cells together with NK cells including subtypes were analyzed in parallel. A total of 108 patients with hematological malignancies transplanted with HLA-matched, T cell replete stem cell grafts were included for analyses of absolute concentrations of CD3, CD4, and CD8 positive T cells and NK cells together with a multi-color flow cytometry panel with staining for TCRαβ, TCRγδ, Vδ1, Vδ2, CD3, CD4, CD8, HLA-DR, CD196, CD45RO, CD45RA, CD16, CD56, CD337, and CD314 at 28, 56, 91, 180, and 365 days after transplantation. Immune reconstitution data including subsets and differentiation markers of T and NK cells during the first year after transplantation was provided. Patients with TCR γδ cell concentrations above the median value of 21 (0–416) × 10^6^ cells/L 56 days after transplantation had significantly improved overall survival (*p* = 0.001) and relapse-free survival (*p* = 0.007) compared to patients with concentrations below this value. When day 56 cell subset concentrations were included as continuous variables, TCR γδ cells were the only T cell subsets with a significant impact on OS and RFS; the impact of TCR γδ cells remained statistically significant in multivariate analyses adjusted for pre-transplant risk factors. The risk of death from relapse was significantly decreased in patients with high concentrations of TCR γδ cells 56 days after transplantation (*p* = 0.003). Also, the risk of acute GVHD was significantly lower in patients with day 28 TCR γδ cell concentrations above the median of 18 × 10^6^ cells/L compared to patients with low concentrations (*p* = 0.01). These results suggest a protective role of TCR γδ cells in relapse and GVHD and encourage further research in developing adaptive TCR γδ cell therapy for improving outcomes after HSCT.

## Introduction

Allogeneic hematopoietic stem cell transplantation (HSCT) is a potentially curative treatment for patients with hematological malignancies by elimination of recipient tumor cells by reactive immune cells from a donor (1). The graft-vs.-leukemia (GVL) effect is originally thought to be mediated by conventional CD4 and CD8 positive T cells expressing the αβ T cell receptor (TCR) through alloreactivity caused by differences in human leukocyte antigens (HLA) and minor histocompatibility antigens (mHAg) between the recipient and the donor (2). When the alloreaction from the donor is aimed at non-malignant recipient tissue, graft-vs.-host disease (GVHD) with extensive morbidity and mortality to the patient can occur (3). The αβ T cells, which constitute the majority of human T cells, raise an adaptive immune response including antigen-specific effector- and memory phases. In contrast, CD4/CD8 negative TCR γδ bearing T cells, which normally constitute about 1–10% of the total T cell repertoire in peripheral blood, operate in an innate-like manner in the initiation phase of the immune reaction independently from HLA-antigen presentation (4). The γδ TCR heterodimer is encoded by two distinct set of genes, γ and δ, with a restricted repertoire of V and J gene segments used for TCR rearrangement. In cord blood the Vδ1 subset constitutes the majority of TCR γδ cells, but already by 1 year of life the Vδ2 subtype becomes the predominant subset in circulation. In adults, the TCR Vδ2 subtype constitutes the majority (>75%) of circulating TCR γδ cells, while tissue- and epithelial associated TCR γδ cells are mainly of the non-Vδ2 (primarily Vδ1) subtype (5). The peripheral Vδ2 subset expand clonally until about 10 years of age and is believed to be driven both by environmental changes and endogenous stimuli in the maturing immune system. TCR γδ cells are capable of tumor-recognition through the γδ TCR and activating receptors shared with natural killer (NK) cells (e.g., NKG2D) (6), and their anti-tumor effects are increasingly being documented in solid tumors as well as in leukemia (7, 8). In the setting of HSCT, the anti-leukemic properties together with the innate-like effector mechanisms in the absence of HLA-restriction suggest that TCR γδ cells mediate GVL in the absence of GVHD (9, 10).

Early immune reconstitution is essential for clinical outcomes after transplantation (11), but recent results on the impact of TCR γδ cell immune reconstitution are primarily from studies in pediatric, HLA-mismatched, and/or graft-manipulated HSCT settings (12–14). Here, we report the results from a prospective, clinical study on the impact of TCR γδ T cell immune reconstitution on overall survival (OS), relapse-free survival (RFS), relapse, and GVHD after HSCT in adult patients receiving HLA-matched, T cell replete stem cell grafts.

## Materials and Methods

### Patients

From October 2015 to March 2017, 113 consecutive patients were transplanted at the Stem Cell Transplantation Unit, Department of Hematology, Copenhagen University Hospital, Rigshospitalet. Two patients declined entrance in the study, the remaining 111 were included. Only patients above 18 years receiving bone marrow (BM) or peripheral blood stem cells (PBSC) and undergoing their first transplantation were included. The study was approved by the Danish National Committee on Health Research Ethics, and all participants gave written informed consent prior to transplantation in accordance with the Declaration of Helsinki. Three patients with non-malignant diseases were later excluded from analyses, Table 1 shows transplant characteristics for the remaining 108 patients.

**Table 1:** Patient and transplant characteristics.

*N*	108
Follow-up time, days, median, range	673 (386–913)
Age, years, median, range	58 (20–74)
**Disease, *n*, percent**	
AML	46 (43%)
ALL	14 (13%)
MDS	25 (23%)
Myelofibrosis	8 (7%)
Lymphoma	6 (6%)
Chronic leukemia	4 (4%)
Other	5 (4%)
**Disease risk index, *n*, percent**	
Low	10 (9%)
Intermediate	87 (81%)
High	11 (10%)
**Graft source, *n*, percent**	
BM	15 (14%)
PBSC	93 (86%)
**Donor, *n*, percent**	
MRD	26 (24%)
MUD	82 (76%)
**Donor match, *n*, percent**	
10/10 or 9/10	97 (90%)
1 Ag MM	9 (8%)
Haplo-identical	2 (2%)
**CMV serological status, D/R**	
+/+	41 (38%)
+/–	8 (7%)
–/–	26 (24%)
–/+	33 (31%)
**Recipient-donor sex, *n*, percent**	
M/M	52 (48%)
M/F	28 (26%)
F/F	17 (16%)
F/M	11 (10%)
**Conditioning intensity, *n*, percent**	
Myeloablative	51 (47%)
Non-myeloablative	57 (53%)
**Conditioning regimen, *n*, percent**	
TBI-Flu	53 (49%)
Flu-Treo	27 (25%)
TBI-Cy	21 (19%)
TBI-Etopophos	4 (4%)
Other	3 (3%)

*AML, acute myeloid leukemia; MDS, myelodysplastic syndrome; ALL, acute lymphoblastic leukemia; BM, bone marrow; PBSC, peripheral blood stem cells; MDR, matched related donor; MUD, matched unrelated donor; TBI, total body irradiation; Flu, fludarabine; Treo, treosulfan; Cy, cyclophosphamide; CMV, cytomegalovirus*.

### Conditioning Regimen

Myeloablative regimens were cyclophosphamide 120 mg/kg plus 12 Gy total body irradiation (TBI); cyclophosphamide was replaced with Etophophos 1,800 mg/m^2^ for acute lymphoblastic leukemia. Cyclophosphamide doses were calculated using adjusted ideal body weight if BMI was >27.5. Non-myeloablative conditioning was fludarabine 90 mg/m^2^ plus 2 Gy TBI; TBI was increased to 4 Gy in patients not previously treated with chemotherapy. High risk MDS patients were conditioned with fludarabine 150 mg/kg plus treosulfan 42 g/mq. The conditioning regimen for haplo-identical transplantation was cyclophosphamide 29 mg/kg, fludarabine 150 mg/m^2^ and 2 Gy TBI. Twelve patients received anti-thymocyte-globulin (ATG) as part of the conditioning regimen: two patients with antigen mismatch received Thymoglobulin 2.5 mg/kg at day −3, −2, and −1, 10 patients with matched unrelated donors and transplanted with PBSC after myeloablative conditioning received Grafalon 10 mg/kg at day −3, −2 and −1.

### GVHD Prophylaxis and Diagnosis

Myeloablative patients received cyclosporine 6.25 mg/kg orally b.i.d. from day −1 combined with a short-course of intravenous methotrexate on days 1 (15 mg/m^2^), 3, 6, and 11 (10 mg/m^2^). Cyclosporine was tapered to stop at day 180, unless GVHD was present. Tacrolimus was used in patients receiving the fludarabine/treosulfan regimen. Non-myeloablative patients received tacrolimus 0.06 mg/kg orally b.i.d. from day −3 combined with mycofenolate mofetil 15 mg/kg b.i.d. from day 0 to days 27 in related transplants. In unrelated transplants, tacrolimus 0.06 mg/kg orally b.i.d from day −3 was combined with mycophenolate mofetil 15 mg/kg orally t.i.d. from day 0 to day 30, then b.i.d. to day 40 and tapered to stop day 96 in the absence of GVHD. In the absence of GVHD, tacrolimus was tapered from day 56 to zero by day 180 in related non-myeloablative transplants, and from day 100 to zero by day 180 in unrelated non-myeloablative transplants. In mismatched non-myeloablative transplants, cyclosporine 5 mg/kg orally b.i.d. and sirolimus 2 mg orally q.d. from day −3 and mycofenolate mofetil 15 mg/kg t.i.d. from day 1 was administered. Cyclosporine was tapered to stop day 180 and mycofenolate mofetil day 150; sirolimus was tapered to stop day 365, unless GVHD was present. In haplo-identical transplants, cyclophosphamide 50 mg/kg was administered day 3 and 4; tacrolimus 1 mg q.d. and mycofenolate mofetil 15 mg/kg t.i.d from day 0 to 35.

Acute and chronic GVHD (aGVHD, cGVHD) was diagnosed and graded from clinical symptoms and biopsies according to the modified Glucksberg-Seattle criteria (15, 16).

### Relapse

Relapse was morphologically diagnosed in leukemia patients as more than 5% blast cells in the bone marrow or the appearance of extramedullary leukemic lesions, in MDS patients, recurrence of MDS by morphology, cytogenetics, or both. Relapse in lymphoma patients was defined by new or progressing foci in PET/CT scans.

### CMV Monitoring and Treatment

Analyses for CMV DNA was done weekly on peripheral blood with quantitative PCR from day 21–100 after transplantation. Cut-off level was 300 CMV DNA copies/ml. In case of a positive CMV PCR, treatment with oral valganciclovir 900 mg b.i.d. was commenced. Patients not able to take oral medication were treated with intravenously ganciclovir 90 mg/kg b.i.d.

### Patient Samples

Peripheral blood samples for evaluation of immune reconstitution were collected at 5 fixed time points within the first year after transplantation: day 28 (median 28 [23–39]), 56 (median 56 [48–76]), 91 (median 91[74–122]), 180 (median 181[148–239]), and 365 (median 364 [334–452]). Samples were collected on 2 mL ethylenediamine tetra acetic tubes and analyzed freshly at the Tissue Typing Laboratory, Department of Clinical Immunology, Copenhagen University Hospital, Rigshospitalet.

### Flow Cytometry

Routine analyses for absolute concentrations of CD3, CD4, and CD8 positive T cells and NK cells were evaluated by flow cytometric analyses with BD™ Trucount tubes containing fluorescent beads as an internal standard according to the manufacturer's instructions (BD Biosciences, San Jose, California). Immunolabeling with CD4 FITC/CD8 PE/CD3 PerCP and CD3 FITC/CD16+CD56 PE/CD45 PerCP from BD™ Biosciences were used. Samples were analyzed on Navios™ (Beckman Coulter, Miami, Florida), and Navios™ software was used for software analyses. Residual blood volume was used for immune phenotyping in a 2-tube, stain-lyse, multi-color flow cytometry panel developed for the study. For multi-fluorochrome staining, 100 μL of whole blood was labeled for TCRαβ-FITC, TCRγδ-PE, CD4-PerCP-Cy5.5, CD45RA-PE-Cy7, CD197-APC, CD45RO-APC-H7, HLA-DR-V450, CD3-V500, and CD8-BV605 for tube 1, and TCRVδ2-FITC, TCRγδ-PE, TCRVδ1-PE-Cy7, CD314-APC, CD16-APC-H7, CD56-V450, CD3-V500, and CD337-BV605 for tube 2. After 15 min incubation, red blood cells were lysed by the addition of 2 mL of lysing solution (EasyLyse™) followed by 10 min of dark incubation at room temperature. This was followed by a 5-min wash procedure (300 g) with 2 mL BD™ FacsFlow and resuspension in 300 mL BD ^TM^ FacsFlow prior to immediate acquisition.

### Acquisition and Data Analysis

Sample acquisition was performed using BD™ FACSCanto flow cytometer with the BD™ FACSDiva software which was also used for data analyses. Phenotype subset definition and gating strategies are shown in Tables 1, 2 and Figure 1 in Supplemental Data.

**Table 2:** T and NK cell concentrations and subset distribution 28–365 days after transplantation.

**Cell subset concentration, 10^6^/L, median (IQR)**	**Day 28 *N =* 106**	**Day 56 *N =* 104**	**Day 91 *N =* 99**	**Day 180 *N =* 86**	**Day 365 *N =* 66**
T cells (CD3)	420 (225–553)	425 (260–738)	460 (240–750)	835 (458–1400)	1150 (680–2000)
CD4	190 (110–300)	190 (113–280)	210 (100–300)	280 (170–420)	395 (250–490)
CD8	150 (71–240)	175 (100–395)	230 (100–440)	440 (198–950)	650 (340–1425)
TCR γδ	18 (6.5–44)	21 (7.2–50)	21 (6.6–53)	36 (12–87)	62 (19–111)
Vδ1	1.5 (0.7–3.4)	1.6 (0.6–4.6)	1.6 (0.6–6.8)	4.4 (1.6–24)	7.9 (3.3–35)
Vδ2	13 (4.8–42)	17 (5.1–39)	14 (4.0–38)	16 (6.1–53)	24 (7.2–62)
NonVδ1-nonVδ2	0.4 (0.2–0.8)	0.3 (0.1–1.0)	0.5 (0.2–1.3)	0.8 (0.3–4.4)	1.2 (0.5–7.3)
NK cells (CD16/56)	280 (140–403)	190 (110–330)	160 (100–270)	240 (130–388)	235 (150–360)
CD16bright	26 (14–60)	25 (12–51)	24 (10–47)	25 (13–55)	27 (15–56)
CD16/56	158 (82–258)	113 (69–202)	106 (65–184)	152 (97–282)	179 (112–294)
CD56bright	57 (27–96)	31 (17–63)	23 (14–38)	17 (12–32)	17 (10–27)
**Relative concentrations, %**					
CD4/CD3	56 (44–65)	49 (28–61)	46 (25–57)	37 (22–50)	35 (23–48)
CD8/CD3	37 (31–52)	45 (36–65)	51 (34–68)	59 (46–74)	62 (47–75)
CD4/CD8, ratio	1.5 (0.9–2.1)	1.0 (0.4–1.8)	0.9 (0.4–1.5)	0.6 (0.3–1.1)	0.6 (0.3–1.0)
Naïve CD4/CD4	28 (15–37)	26 (12–37)	26 (15–35)	21 (12–32)	22 (13–29)
Central memory CD4/CD4	40 (34–49)	41 (33–49)	40 (34–48)	40 (31–48)	43 (34–48)
Effector memory CD4/CD4	25 (19–37)	29 (18–42)	27 (19–39)	32 (22–46)	31 (23–42)
TEMRA CD4/CD4	0.8 (0.4–1.8)	1.1 (0.6–2.5)	1.3 (0.6–2.5)	1.5 (0.9–4.8)	1.7 (0.9–4.7)
HLA-DR CD4/CD4	21 (13–40)	27 (17–51)	31 (21–49)	37 (24–51)	26 (20–37)
Naïve CD8/CD8	23 (7.6–37)	13 (4.4–33)	13 (5.8–26)	12 (5.9–22)	9.4 (4.4–19)
Central memory CD8/CD8	4.7 (2.9–7.9)	4.3 (2.3–8.4)	5.2 (2.9–9.2)	5.6 (3.1–9.5)	5.6 (2.6–9.2)
Effector memory CD8/CD8	39 (27–56)	40 (29–52)	36 (28–47)	32 (24–43)	37 (29–52)
TEMRA CD8/CD8	21 (13–38)	32 (18–45)	36 (24–52)	44 (33–57)	39 (23–51)
HLA-DR CD8/CD8	40 (22–61)	52 (26–75)	58 (40–74)	64 (50–78)	52 (35–62)
Naïve TCR γδ/TCR γδ	1.8 (0.9–4.0)	1.6 (0.6–3.4)	2.1 (0.8–4.3)	2.0 (1.0–4.5)	2.1 (0.7–4.5)
Central memory TCR γδ/TCR γδ	1.2 (0.4–2.3)	0.9 (0.3–2.6)	0.9 (0.2–2.2)	1.2 (0.3–3.5)	2.1 (0.6–4.0)
Effector memory TCR γδ/TCR γδ	51 (37–67)	48 (32–61)	38 (23–54)	37 (22–52)	43 (27–65)
TEMRA TCR γδ/TCR γδ	46 (29–61)	49 (35–65)	52 (40–69)	58 (38–72)	47 (25–63)
HLA-DR TCR γδ/TCR γδ	44 (28–58)	40 (22–57)	45 (30–61)	47 (33–68)	36 (25–53)
TCR γδ/CD3 T	4.6 (2.6–11)	4.5 (2.3–9.8)	4.1 (2.0–10)	3.7 (2.4–7.4)	4.0 (2.1–7.6)
Vδ1/TCR γδ	10 (3.3–24)	11 (3.7–22)	13 (3.8–30)	28 (8.5–48)	35 (10–56)
Vδ2/TCR γδ	88 (70–95)	86 (68–95)	82 (60–95)	67 (38–90)	61 (31–86)
NonVδ1-nonVδ2/TCR γδ	2 (0.8–5.8)	2.1 (0.6–5.2)	3.0 (0.9–7.8)	3.9 (0.9–9.6)	4.0 (1.1–12)
CD16bright/NK	11 (6.2–24)	14 (7.7–21)	15 (8–22)	12 (7.8–21)	13 (7.3–19)
CD16CD56/NK	59 (47–70)	68 (54–77)	68 (55–81)	76 (65–83)	80 (71–86)
CD56bright/NK	23 (16–33)	15 (10–24)	13 (8.3–22)	8.7 (5.1–14)	7.4 (3.7–13)

**Figure 1:**
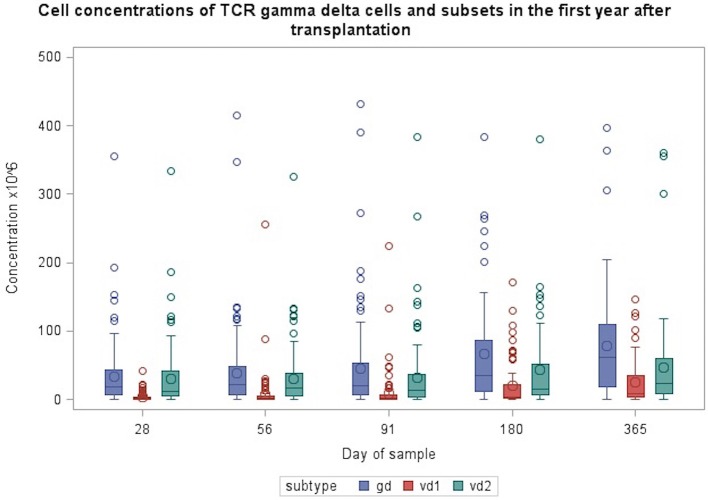
Concentrations of TCR γδ cells and Vδ1 and Vδ2 subsets during the first year after transplantation. Patient numbers day 28 *n* = 106, day 56 *n* = 104, day 91 *n* = 99, day 180 *n* = 86, day 365 *n* = 66 (values from one patient with day 180 TCR γδ cell concentrations of 632 mio/L and Vδ2 concentration of 570 mio/L are not included in the figure).

### Immune Reconstitution Analyses

Analyzed lymphocyte subset were absolute concentrations of total TCR γδ cells, TCR Vδ1, TCR Vδ2, CD3 T cells, CD4 T cells, CD8 T cells, total NK cells, CD16bright NK cells, CD16/56 NK cells, and CD56bright NK cells. In addition, we analyzed fractions of differentiation subsets in terms of naïve (CD45RA+CD197+), central memory (CD45RA–CD197+), effector memory (CD45RA–CD197–) and TEMRA (CD45RA+CD197–) cells of CD4, CD8, and TCR γδ cells. The fractions of TCR γδ cells of total CD3 cells, the Vδ1, Vδ2, and nonVδ1-nonVδ2 of total TCR γδ cells, and the CD16bright, CD16/56, and CD56bright of total NK cells were also analyzed. The expression of HLA-DR as a marker of activation was analyzed on total TCR γδ cells, CD4 T cells, and CD8 T cells. The expression of the activating receptor NKG2D was analyzed on total TCR γδ cells, Vδ1, and Vδ2 cells. Throughout the text, “concentration” refers to absolute concentrations (10^6^/L) and “percentages” or “fractions” refer to percent of the specified cell subsets of the specified cell populations. Cell concentrations were analyzed as continuous and categorical variables (high vs. low) dichotomized by the median value of the above-mentioned absolute cell concentrations.

### Outcomes

The primary outcomes were overall survival (OS) and relapse-free survival (RFS) from day 56. OS was defined as the probability of survival from day 56 with death as an event. RFS survival was defined as the probability of survival without relapse from day 56 with an event defined as the composite of death and/or relapse. Day 56 after transplantation was selected for the primary outcome as this was the closest time point to relapse occurrence, which still preceded relapse in all patients. Secondary outcomes included death from relapse, aGVHD and cGVHD. For associations to aGVHD the earliest sample after transplantation (day 28) was used. Nine patients were diagnosed with aGVHD before their respective day 28 sample and were therefore excluded from the aGVHD analyses. Association to cGVHD was performed for both day 28 and day 56 immune reconstitution. Furthermore, associations between post-transplant CMV infection and TCR γδ cell immune reconstitution (high vs. low median concentrations and fractions) were analyses at day 56, 91, 180, and 365 after transplantation. For each time point only patients with CMV infection diagnosed at least 1 week prior to their blood sampling were included for time to establish an immunological cellular response (for this reason associations with day 28 immune reconstitution were not analyzed, as only 3 patients had CMV infection more than 1 week before their respective day 28 sample). The associations between pre-transplant CMV status of the donor and TCR γδ cell immune reconstitution were tested in all patients and for all time points after transplantation. For all outcomes, patients with graft rejection (*n* = 1) and graft failure (*n* = 2) were censored at the time of rejection or booster transplantation.

### Statistical Analyses

Kaplan Meier survival analysis and Cox proportional hazards models were used to investigate the associations between immune reconstitution and OS and RFS. In addition to cell concentrations, pre-transplant factors thought to have a possible impact on OS and RFS were included in the analysis. Disease Risk Index was included for all patients after previously published criteria (17). Due to size of the dataset only factors distributed with more than 10% of patients in the smallest group were included. Pre-transplant factors with a *p* < 0.1 in univariate analysis were included in the multivariable models. The pre-transplant factors were analyzed in patients alive by day 28 after transplantation (*n* = 106) and these results were used for all multivariate analyses.

The cumulative incidence of aGVHD and death from relapse, was determined using Gray's competing risks analysis. Competing risk for death from relapse was death from transplant-related mortality (TRM). TRM was defined as death from all causes other than relapse. Competing risk for aGVHD was death from all causes other than aGVHD. Only grade II-IV aGVHD were included in the aGVHD analyses. Associations between CMV status of the donor/post-transplant CMV infection and TCR γδ cell immune reconstitution were analyzed by the χ^2^-test. Differences between categorical and continuous variables were determined by the χ^2^ and Student's *T*-test, respectively. Spearman correlation was used for non-parametric assessment of correlation between different cell subsets during immune reconstitution.

### Sensitivity Analysis

In sensitivity analysis Cox-proportional hazards models were used to the investigate the robustness of the results when cell concentrations were included as time-update variables. For these analyses all patients with a sample at day 28 were included from the date of that sample and followed until relapsed, death or the last follow-up date, depending on the outcome of interest.

Statistical analyses were performed using SPSS version 22 (SPSS, Chicago, IL) and R version 3.2.0 (R Foundation for Statistical Computing, Vienna, Austria) combined with the EZR platform (18) and SAS version 9.4.

## Results

### Patient Outcome

Patients were followed for a median of 673 (386–913) days after transplantation. At the end of follow-up 80 (74%) of the 108 patients were alive; 14 (13%) patients died from TRM and 14 (13%) died from relapse. Overall survival in all patients was 61% (95% CI 35–79%), RFS was 47% (95% CI 24–68%), and TRM was 23% (95 CI 4.7–50%). In the TRM group, the registered cause of death was aGVHD in four patients, cGVHD in one patient, infection in three patients, organ failure in three patients, toxicity in one patient, and lung embolism in one patient. One patient died from unknown causes other than relapse later than 2 years post-transplant. Two and four patients died from TRM prior to their day 28/56 samples. A total of 24 (22%) experienced relapse during the follow-up time with a median time to relapse of 177 (56–778) days. Acute GVHD grade II-IV was diagnosed in 38 (35%) patients, and cGVHD in 54 (50%) patients. A total of 28 patients (26%) experienced CMV infection at a median of 42 (15–101 days) after transplantation.

### Immune Reconstitution

Table 2 shows concentrations and subset fractions of T cells and NK cells 28, 56, 91, 180, and 365 days after transplantation. Median concentrations of CD3 cells doubled after 6 months and tripled after 1 year; the CD4/CD8 ratio shifted from 1.5 to 0.6 as the concentration of CD8 cells increased more than 4-fold during the first year post-transplant. The total NK cell concentration was highest early after transplantation (day 28) and decreased until 3 months after transplantation; hereafter it increased by 6 and 12 months post-transplant. The CD56bright NK subset peaked early after transplantation and declined steadily during the first year.

**Figure 2:**
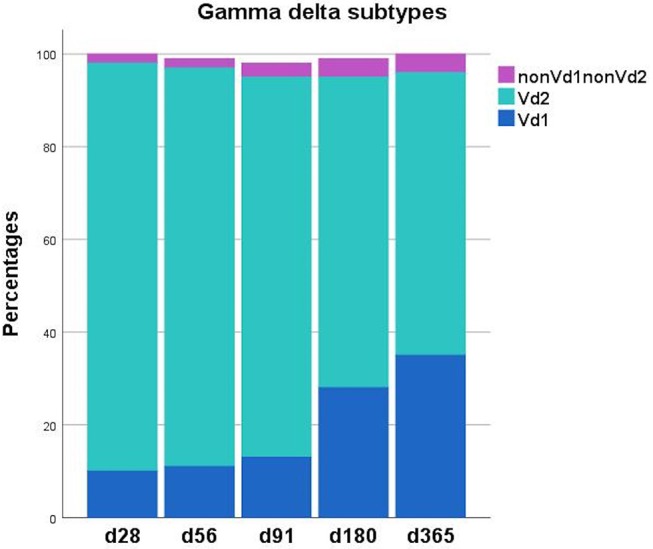
Distribution of TCR γδ T cell subsets (Vδ1, Vδ2, nonVδ1-nonVδ) during the first year after transplantation. Each compartment is constructed by the use of median percentages. Patient numbers day 28 *n* = 106, day 56 *n* = 104, day 91 *n* = 99, day 180 *n* = 86, day 365 *n* = 66.

Table 2, Figures 1, 2 shows immune reconstitution of TCR γδ cell concentrations and subset fractions during the first year after transplantation; median TCR γδ cell concentrations remained relatively constant within the first 3 months, doubled after 6 months and tripled after 12 months. The fraction of TCR γδ cells of the total CD3 T cell concentration remained stable below 5%, while the TCR Vδ1 fraction of the total TCR γδ cell concentration increased, and the TCR Vδ2 fraction decreased during the first 12 months after transplantation, Figure 2.

**Figure 3:**
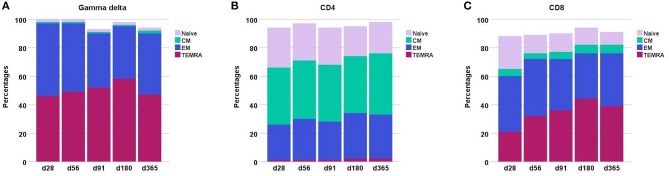
Differentiation subsets in **(A)** the TCR γδ compartment **(B)** the CD4 compartment, and **(C)** the CD8 compartment during the first year after transplantation. The fractions are constructed with the use of median percentages. Patient numbers day 28 *n* = 106, day 56 *n* = 104, day 91 *n* = 99, day 180 *n* = 86, day 365 *n* = 66. CM, central memory; EM, effector memory.

Throughout the first year after transplantation the total TCR γδ cell compartment consists mainly of effector memory and TEMRA cells, Figure 3. In contrast, the CD8 T cell and especially the CD4 T cell compartment is constituted by much higher fractions of naïve cells. Moreover, the CD4 compartment contains a substantially higher percentage of central memory cells compared to both the CD8 and TCR γδ cell compartments. The distribution of the subsets remained relatively constant through the first year after transplantation in the CD4 T cells compartment while the most striking change occurred in the decreasing percentages of naïve cells in the CD8 compartment. The percentages of TEMRA cells increased during the first 6 months in both the TCR γδ cell and CD8 T cell compartment.

Median TCR γδ cell concentration 56 days after transplantation was 21 (IQR 7.2–50) × 10^6^/L, Table 2. A higher proportion of patients with high TCR γδ cell concentrations at day 56 had donor age <30 years (67 vs. 56%), 9–10/10 allele HLA-match (96 vs. 83%), AML/MDS disease type (75 vs. 56%), and received PBSC as a graft source (94 vs. 77%), as shown in Table 3. Of note, the use of ATG in the conditioning regimen was not significantly different in patients with high TCR γδ cell concentration compared to low. The TCR γδ cell concentration day 56 was significantly correlated to both CD3, CD4, and CD8 T cells and NK cell concentrations, and these were also significantly correlated to each other (*p* < 0.01 for all comparisons, data not shown).

**Table 3:** Distribution of pre-transplant factors associated with TCR γδ cell concentrations 56 days after transplantation (χ^2^).

	**Total**	**Day 56 TCR γδ high (>21 × 10^**6**^/L)**	**Day 56 TCR γδ low (<21 × 10^**6**^/L)**	***P*-value**
*N*	104	52	52	
**Age**				
≤45	23	13 (57%)	10 (43%)	0.48
>45	81	39 (48%)	42 (52%)	
**Disease type**				
AML/MDS	68	39 (57%)	29 (43%)	*0.04*
Other	36	13 (36%)	23 (64%)	
**Donor age**				
<30	59	35 (59%)	24 (41%)	*0.03*
>30	45	17 (38)	28 (62%)	
**Disease stage**				
Early	59	28 (47%)	31 (53%)	0.55
Intermediate/Late	45	24 (53%)	21 (47%)	
**Recipient CMV serological status**				
Positive	71	35 (49%)	36 (51%)	0.83
Negative	33	17 (52%)	16 (48%)	
**Donor CMV serological status**				
Positive	46	18 (39%)	28 (61%)	0.08
Negative	58	34 (59%)	24 (41%)	
**Donor-recipient sex**				
Female-male	10	4 (40%)	6 (60%)	0.51
Other	94	48 (51%)	46 (49%)	
**Donor type**				
MRD	21	11 (52%)	10 (48%)	0.36
MUD	81	41 (51%)	40 (49%)	
Haplo-identical	2	0 (0%)	2	
**HLA-match**				
9/10 or 10/10 allele match	93	50 (54%)	43 (46%)	*0.03*
Other (antigen MM+haplo-identical)	11	2 (18%)	9 (82%)	
**Stem cell source**				
BM	15	3 (20%)	12 (80%)	*0.01*
PBSC	89	49 (55%)	40 (45%)	
**Conditioning regimen**				
Myeloablative	48	26 (54%)	22 (46%)	0.43
Non-myeloablative	56	26 (46%)	30 (54%)	
**ATG**				
Yes	11	6 (55%)	5 (45%)	0.75
No	93	46 (49%)	47 (51%)	

*MDR, matched related donor; MUD, matched unrelated donor; MM, mismatch; BM, bone marrow; PBSC, peripheral blood stem cells; ATG, anti-thymocyte-globulin. The italic values represent p-values equal to or below 0.05*.

### Immune Reconstitution and Clinical Outcomes

Table 4 shows the results of the univariate analysis investigating the association between pre-transplant factors and death. Donor age >30 years (HR 2.12, 95% CI 0.98–4.57) and non-myeloablative conditioning regimen (HR 2.23, 95% CI 0.98–5.10) were associated with an increased risk of death. The same factors were associated with an increased risk of death or relapse and thus were included in the multivariate models investigating the association with cell concentrations and OS/RFS.

**Table 4:** Univariate analyses of pre-transplant factors and the impact on overall survival and relapse-free survival from day 28, *n* = 106.

**Variable**	***N***	**Overall survival**		**Relapse-free survival**	
		**HR (95% CI)**	***P*-value**	**HR (95%CI)**	***P*-value**
**Recipient age**					
Per 10 years older	–	1.15 (0.84–1.59)	0.39	1.04 (0.80–1.36)	0.76
**Disease type**					
AML/MDS	70	1.00	0.38	1.00	0.36
Other	36	1.42 (0.66–3.06)		1.37 (0.70–2.68)	
**Donor age**					
<30 years	60	1.00		1.00	
>30 years	46	2.12 (0.98–4.57)	0.06	2.58 (1.32–5.04)	0.01
**Donor type**					
Matched related donor	23	1.00		1.00	
Matched unrelated donor	83	1.50 (0.52–4.36)	0.45	0.83 (0.38–1.84)	0.65
**Graft source**					
PBSC	91	1.00		1.00	
BM	15	0.99 (0.34–2.87)	0.99	0.88 (0.34–2.26)	0.79
**Conditioning regimen**					
Myeloablative	49	1.00		1.00	
Non-myeloablative	57	2.23 (0.98–5.10)	0.06	1.80 (0.92–3.55)	0.09
**ATG**					
No	94	1.00		1.00	
Yes	12	0.69 (0.16–2.93)	0.62	1.51	0.40
**CMV donor**					
Negative	58	1.00		1.00	
Positive	48	1.60 (0.75–3.43)	0.22	1.43 (0.75–2.74)	0.28
**CMV recipient**					
Negative	33	1.00		1.00	
Positive	73	0.88 (0.40–1.97)	0.76	0.88 (0.44–1.76)	0.72
Disease risk index^*^					
Low	10			1.00	
Intermediate	85			5.05 (0.69–36.98)	
High	11	–		4.11 (0.46–36.92)	0.27

Hazard ratios are of death in overall survival and of death or relapse in relapse-free survival. AML, acute myeloid leukemia; MDS, myelodysplastic syndrome; CMV, cytomelagovirus; DRI, Disease Risk Index.

* *Disease Risk Index, as there were no deaths in the low risk group during the observation time, no estimate of this variable is included for OS*.

### Overall Survival and Relapse-Free Survival

**Figure 4:**
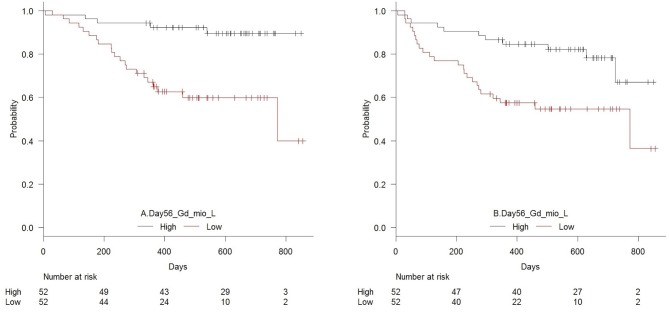
Estimated **(A)** overall survival (*p* = 0.001) and **(B)** relapse-free survival (*p* = 0.007) in patients with high vs. low concentrations (median 21 × 10^6^/L) of TCR γδ cells 56 days after transplantation, *n* = 104.

Figure 4 shows OS and RFS for patients with high vs. low day 56 median concentrations of TCR γδ cells. In the T cell compartment, patients with low (below the median) concentrations of TCR γδ cells, TCR Vδ1 cells, TCR Vδ2 cells, and CD4 cells 56 days after transplantation had an increased risk of death compared to patients with high concentrations, Table 5A. Within the NK cell compartment, low median concentrations of the CD56bright subset was significantly associated with increased risk of death. These cell subtypes remained significantly associated with death after adjustment for pre-transplant factors (donor age and conditioning regimen). Patients with low TCR γδ cells at day 56 had over a 5 times greater risk of death compared to those with TCR γδ cells above the median (HR 5.16, 95% CI 1.94–13.7, *p* = 0.001) Similar associations were observed for relapse free survival where patients with low concentrations of TCR γδ cells at day 56 had a 2.7 (95% CI 1.32–5.53, *p* = 0.007) times greater risk of death or relapse compared to patients with high concentrations, this also remained significant after adjustment for pre-transplant factors, as indicated in Table 5A.

**Table 5:** Univariate analyses of the impact of day 56 cell subsets on overall survival and relapse-free survival in **(A)** cell subsets included with median cut-off values and **(B)** cell subsets included as continuous (log_2_-transformed) values, *n* = 104.

**(A)**				
**Cell subset**	**Overall survival**		**Relapse-free survival**	
	**HR (95% CI)**	***P*** **=** **value**	**HR (95% CI)**	***P*** **=** **value**
CD3				
High	1.00		1.00	
Low	2.18 (0.97–4.90)	0.06	1.15 (0.77–2.96)	0.23
CD4				
High	1.00		1.00	
Low	2.73 (1.15–6.48	0.02^*^	1.99 (0.99–4.00)	0.05^*^
CD8				
High	1.00		1.00	
Low	2.12 (0.94–4.76)	0.07	1.45 (0.74–2.84)	0.28
TCR γδ				
High	1.00		1.00	
Low	5.16 (1.94–13.7)	0.001^**^	2.70 (1.32–5.53)	0.007^*^
Vδ1				
High	1.00		1.00	
Low	2.26 (1.00–5.01)	*0.05*	2.20 (1.09–4.45)	0.03^*^
Vδ2				
High	1.00		1.00	
Low	5.02 (1.89–13.4)	0.001^**^	2.67 (1.30–5.50)	0.007^*^
NK				
High	1.00		1.00	
Low	1.55 (0.7–3.43)	0.28	1.98 (0.98–3.98)	0.06
CD16b				
High	1.00		1.00	
Low	1.96 (0.87–4.42)	0.11	1.35 (0.69–2.66)	0.38
CD16/56				
High	1.00		1.00	
Low	2.01 (0.89–4.54)	0.09	2.14 (1.06–4.32)	0.03^*^
CD56b				
High	1.00		1.00	
Low	2.31 (1.00–5.34)	0.05^*^	1.81 (0.91–3.61)	0.09
**(B)**				
**Cell subset, per doubling value**	**Overall survival**		**Relapse-free survival**
	**HR (95% CI)**	***P*** **=** **value**	**HR (95% CI)**	***P*** **=** **value**
CD3	0.85 (0.64–1.42)	0.29	0.88 (0.67–1.14)	0.33
CD4	0.86 (0.64–1.16)	0.33	0.86 (0.67–1.12)	0.27
CD8	0.79 (0.59–1.05)	0.10	0.83 (0.65–1.07)	0.16
TCR γδ	0.63 (0.49–0.80)	<0.001^**^	0.76 (0.61–0.93)	0.008^*^
Vδ1	0.65 (0.44–0.96)	0.03^*^	0.81 (0.60–1.09)	0.16
Vδ2	0.65 (0.51–0.81)	<0.001^**^	0.75 (0.62–0.91)	0.003^*^
NK	0.75 (0.52–1.07)	0.11	0.81 (0.60–1.10)	0.18
CD16b	0.83 (0.63–1.09)	0.18	0.95 (0.75–1.19)	0.65
CD16/56	0.85 (0.60–1.18)	0.33	0.86 (0.6–1.13)	0.28
CD56b	0.61 (0.45–0.84)	0.002^*^	0.74 (0.56–0.97)	0.03^*^

Hazard ratios are of death in overall survival and of death or relapse in relapse-free survival. Cell subsets significant in multivariate analyses including adjustments for pre-transplant factors (conditioning therapy and donor age) are indicated by ^*^,

* *p ≤ 0.05*,

** *p ≤ 0.01. The italic values represent p-values equal to or below 0.05*.

When cell subset concentrations were included as continuous variables, Table 5B, in the T cell compartment, increasing concentrations of TCR γδ cells, TCR Vδ1, and TCR Vδ2 cells were significantly associated with decreased risk of death while increasing concentrations of TCR γδ cells and TCR Vδ2 cells were significantly associated with decreased risk of death or relapse In the NK cell compartment, increasing concentrations of the CD56b subset were significantly associated with both outcomes. All significant cell subsets remained significant in the multivariate analyses adjusted for pre-transplant factors as indicated in Table 5B.

After including a further adjustment for other significant cell subtypes in the multivariable analysis, only median day 56 concentrations of TCR γδ cells remained significant associated with an increased risk of death (HR 4.20, 95% CI 1.55–11.4, *p* = 0.005) and death or relapse (HR 2.26, 95% CI 1.08–4.77, *p* = 0.03), Table 6.

**Table 6:** Multivariate analyses of the impact of day 56 TCR γδ cell concentration (median cut-off values) including other significant cell subtypes, *n* = 104.

**Cell subset**	**Overall survival**		**Relapse-free survival**	
	**HR (95% CI)**	***P*-value**	**HR (95% CI)**	***P*-value**
**TCR γδ cell concentration**				
High	1.00		1.00	
Low	4.20 (1.55–11.4)	*0.005*	2.26 (1.08–4.77)	*0.03*
**CD4 T cell concentration**				
High	1.00		1.00	
Low	1.82 (0.74–4.44)	0.19	1.36 (0.63–2.92)	0.42
**CD16CD56 cell concentration**				
High			1.00	
Low	–		1.64 (0.77–3.49)	0.20
**CD56b cell concentration**				
High	1.00		–	
Low	1.68 (0.72–3.95)	0.23		

*The italic values represent p-values equal to or below 0.05*.

### Death From Relapse

**Figure 5:**
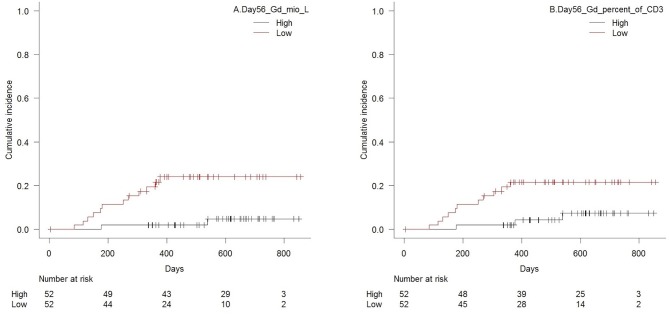
Cumulative incidence (Gray's test for competing risk) of death from relapse with death from transplant-related-mortality as a competing event in patients with high vs. low **(A)** concentrations of TCR γδ cells (median 21 × 10^6^/L), *p* = 0.003, and **(B)** TCR γδ cell fraction of the total CD3 T cell concentration (median 4.5%), *p* = 0.02, 56 days after transplantation, *n* = 104.

Patients with low TCR γδ cell concentrations 56 days after transplantation had a significantly higher cumulative incidence of death from relapse compared to patients with low concentrations, *p* = 0.003 (Figure 5A), and *p* = 0.003 for TCR Vδ2 concentrations separately. High fractions of TCR γδ T cells of the total CD3 T cell concentration was also significantly associated to lower risk of death from relapse, *p* = 0.02, Figure 5B. No significant association was observed between death from relapse and day 56 concentrations of total CD3 cells, CD4, or C8 cells (*p* = 0.67, 0.38, and 0.66, respectively). The only other cell subset concentration significantly associated with lower risk of death from relapse was the CD56bright NK cell subset for high vs. low concentrations (*p* = 0.03). The total median NK cell concentration, the CD16bright subset and the CD16CD56 subset were not associated to death from relapse (*p* = 0.98, 0.96 and 0.49, respectively).

### Sensitivity Analysis

In addition to analyses of the cell subsets concentrations at day 56 post-transplant, cells subsets were analyzed as continuous, time-updated variables. Results from these sensitivity analyses are presented in Table 7 for the 106 patients with a day 28 sample. As shown, increasing concentrations of all cell subsets apart from the CD16b and the CD56b NK cells were significantly associated with a decreased risk of death in both univariate and multivariate analyses. The findings were consistent when relapse-free survival was the considered. Similarly, in univariate analyses of death from relapse, with death from TRM as competing risk, increasing concentrations of all T cell subsets and the NK CD16/56 subset were significantly associated with increased risk of death from relapse (data not shown).

**Table 7:** Univariate analyses of the impact of time-updated cell subset concentrations (log_2_ transformed continuous variables) on overall survival and relapse-free survival, *n* = 106.

**Cell subset**	**Overall survival**		**Relapse-free survival**	
	**HR (95% CI)**	***p*-value**	**HR (95% CI)**	***p*-value**
CD3	0.56 (0.43–0.74)	<0.0001^***^	0.58 (0.45–0.74)	<0.0001^***^
CD4	0.50 (0.37–0.66)	<0.0001^***^	0.52 (0.40–0.68)	<0.0001^***^
CD8	0.64 (0.50–0.81)	0.0002^***^	0.64 (0.52–0.79)	<0.0001^***^
TCR γδ	0.66 (0.53–0.83)	0.0003^***^	0.72 (0.59–0.88)	0.001^**^
Vδ1	0.74 (0.59–0.93)	0.01^*^	0.83 (0.69–1.00)	*0.04*
Vδ2	0.68 (0.54–0.85)	0.0003^**^	0.74 (0.61–0.90)	0.002^**^
NK	0.61 (0.43–0.87)	0.007^**^	0.63 (0.46–0.87)	0.005^**^
CD16b	0.77 (0.58–1.04)	0.09	0.90 (0.70–1.16)	0.41
CD16/56	0.59 (0.42–0.84)	<0.0001^***^	0.59 (0.44–0.81)	0.0008^***^
CD56b	0.78 (0.55–1.10)	0.16	0.94 (0.69–1.29)	0.71

Cell subsets significant in multivariate analyses including adjustments for conditioning therapy and donor age are indicated by ^*^,

* *p ≤ 0.05*,

** *p ≤ 0.01*,

*** *p ≤ 0.001. The italic values represent p-values equal to or below 0.05*.

### Acute GVHD

**Figure 6:**
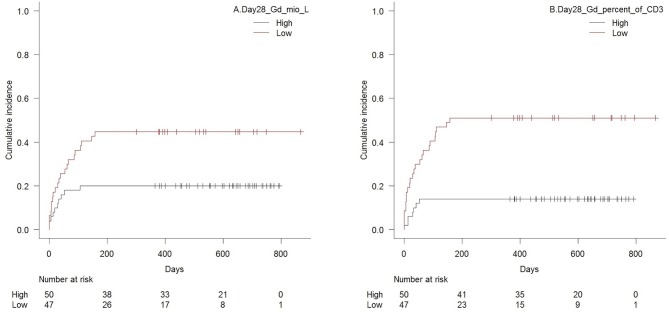
Cumulative incidence (Gray's test for competing risk) of acute GVHD with death as competing event in patients with high vs. low **(A)** concentrations of TCR γδ cells (median 18 × 10^6^/L), *p* = 0.01, and **(B)** TCR γδ cell fraction of the total CD3 T cell concentration (median 4.6%), *p* < 0.001, 28 days after transplantation, *n* = 97. Patients diagnosed with acute GVHD before their respective day 28 sample were excluded.

Patients with high TCR γδ cell concentrations 28 days after transplantation had significantly lower risk of aGVHD with death as competing event compared to patients with low concentrations, *p* = 0.02 for total TCR γδ cells (Figure 6A) and *p* = 0.02 for the TCR Vδ2 subset separately. Association to the TCR Vδ1 subset showed the same trend (*p* = 0.08). Analyzes of differentiation subsets of the total TCR γδ population showed that it was specifically the concentration of effector memory TCR γδ cells that was associated with aGVHD, as patients with values above the median of 8.4 × 10^6^/L had significantly lower risk of developing aGVHD compared to patients with values below this concentration (*p* = 0.001). The strongest association was, however, with high vs. low fractions of TCR γδ cells of the total CD3 T cell concentration, *p* < 0.001, Figure 6B. There were no association between day 28 median concentrations of total CD3 cells (*p* = 0.65), CD4 cells (*p* = 0.71), or CD8 cells (*p* = 0.84) and aGVHD. High concentrations of the CD56bright NK cell subset and the CD16bright NK cell subset 28 days after transplantation were also significantly associated with lower risk of aGVHD, *p* = 0.02 and *p* = 0.01, respectively, while there was a non-significant association to the total NK cell concentration (*p* = 0.06) The CD16/56 subset was not associated with aGVHD (*p* = 0.41).

### Chronic GVHD

TCR γδ cell concentrations on day 28 and 56 were not associated to the risk of cGVHD with death as competing event, *p* = 0.98 and *p* = 0.79, respectively. High CD4 T cell concentrations on both day 28 and 56 were significantly associated with increased risk of cGVHD, *p* = 0.006 and *p* = 0.04, respectively; separate analyses of differentiation subsets of CD4 T cells showed that it was specifically the concentration of naïve CD4 T cells that was associated with cGVHD; patients with values above the median of 58 × 10^6^/L had a trend toward increased risk of developing cGVHD compared to patients with values below this concentration by day 28 (*p* = 0.09) and this association became significant by day 56 (*p* = 0.03) when median concentration of naïve CD4 T cell was 53 × 10^6^/L. None of the other T cell or NK cell subsets showed any significant associations to cGVHD 28 or 56 days after transplantation.

### Differentiation and Activation Markers of TCR γδ Cells Day 56

Since the day 56 TCR γδ cell immune reconstitution was significantly associated with relapse and RFS, we further investigated the possible effect of differentiation and activation of the TCR γδ cells at this time point. The TCR γδ cell compartment 56 days after transplantation in all patients consisted of a median of 1.6% naive cells, 0.9% central memory cells, 48% effector memory cells, and 49% TEMRA cells, Table 2, Figure 3. A median of 40 (IQR 22–57) % of all TCR γδ cells were HLA-DR positive, and >99% of both total TCR γδ cells, Vδ1, and TCR Vδ2 cells were positive for the activating receptor NKG2D. We found no differences in concentrations or fractions of TCR γδ naïve, central memory, effector memory or TEMRA subsets in patients who relapsed compared to patients without relapse. The number of HLA-DR positive TCR γδ cells and the median MFI values of NKG2D expression on total TCR γδ cells, Vδ1, and Vδ2 cells were also not statistically different in patients who relapsed compared to patients without relapse (data not shown). Since >99 percent of the total TCR γδ cells, Vδ1, and Vδ2 cells were positive for the expression of NKG2D, we made no comparison of the numbers of percent positives cells of this value between the groups.

### CMV and Association to TCR γδ Cell Immune Reconstitution

**Figure 7:**
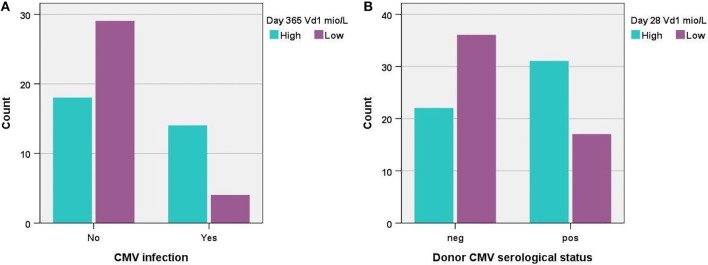
**(A)** Distribution of patients with high vs. low concentrations (median 7.9 × 106/L) of TCR Vδ1 cells 1 year post-transplant according to CMV infection, *p* = 0.004, *n* = 66, and **(B)** distribution of patients with high vs. low concentrations (median 1.5 × 10^6^/L) of TCR Vδ1 cells 28 days post-transplant according to donor serological CMV status, *p* = 0.006, *n* = 106.

We tested the association between post-transplant CMV infection and immune reconstitution of the TCR γδ cell compartment for day 56, 91, 180, and 365 after transplantation. We saw no significant differences in immune reconstitution on day 56 or 91 after transplantation and previous CMV infection. Day 180 and 365 analyses (Figure 7A) however, showed that patients with CMV infection had significantly higher absolute concentrations of Vδ1 cells, significantly higher percentages of Vδd1/TCR γδ cells and significantly lower percentages of Vδ2/TCR γδ cells compared to patients who had not had CMV infection. The percentages of nonVδ1-nonVδ2/TCR γδ cells were also significantly higher in patients with CMV infection at 365 days after transplantation (data not shown). The concentration of total TCR γδ cells was not associated with CMV infection at any time point after transplantation. Regarding differentiation subsets, patients diagnosed with CMV had significantly lower concentrations and percentages of central effector TCR γδ of total TCR γδ cells together with a non-significant trend toward higher concentrations and percentages of TEMRA TCR γδ cells compared to patients without CMV at 180 and 365 days after transplantation (data not shown). One year after transplantation, patients with previous CMV had significantly lower concentrations of naïve TCR γδ cells and a trend toward higher concentrations and fractions of TEMRA TCR γδ cells compared to patients without CMV. Subsequently, we tested if CMV infection was an independent factor associated with relapse incidence and RFS at a landmark analyses 100 days after transplantation (9 patients had events before day 100, one patient diagnosed with CMV day 28 died before day 100). Previously diagnosed CMV infection was not an independent factor associated with relapse incidence (*p* = 0.49 cumulative incidence,) or RFS (*p* = 0.43, KM, log rank) in all patients (*n* = 99). A subgroup of patients with MDS or AML (*n* = 66) was tested separately, also with no association to relapse incidence (*p* = 0.28) or RFS (*p* = 0.51), data not shown.

The serological status of the donor was also tested for associations to immune reconstitution of TCR γδ cells the first year after transplantation. Recipients of CMV positive donors had significantly higher percentages of Vδ1/TCR γδ cells and significantly lower percentages of Vδ2/TCR γδ compared to recipients of CMV negative donors the first 3 months after transplantation (time points day 28, 56 and 91), Figure 7B. The absolute concentrations of Vδ1 and Vδ2 cells were similarly significantly different in these patients 28 days after transplantation, but not at later time points. The percentages of nonVδ1-nonVδ2/TCR γδ cells were significantly higher in recipients of CMV positive donors compared to recipients of CMV negative donors 28 and 56 days after transplantation (data not shown). By day 180 and day 365 after transplantation there was no longer associations to the serological status of the donor. The concentration of total TCR γδ cells was not significantly associated with the serological status of the donor at any time point.

## Discussion

Following earlier, small-numbered studies of the clinical impact of the immune reconstitution of TCR γδ cells with contrasting results (19–21), a 5-year follow-up study in 2007 confirmed improved disease-free survival and overall survival in patients with high concentrations of circulating TCR γδ cells after HLA-mismatched, TCR αβ cell depleted HSCT (12). Recently, a pediatric study demonstrated lower probability of infections and increased event-free survival in patients with high concentrations of TCR γδ cells in a diverse HSCT-setting including both haplo-identical donors and the use of umbilical cord blood as graft source (13). In 2017, a pediatric study demonstrated how infusion of zoledronic acid in recipients of αβ T- and B-cell-depleted HLA-haploidentical HSCT stimulates γδ T-cell differentiation and cytotoxicity and may increase patient survival (22). Adult HLA-matched transplants, however, are by far the most common type of transplant (23), and we here report data from a prospective, clinical study in this setting.

We found a significantly increased OS and RFS with lower risk of relapse and aGVHD in patients with high TCR γδ cell concentrations 56 days after HLA-matched, T cell replete transplantation. The studies by Godder et al. (12) and Perko et al. (13) used the cut-off limit of 150/175 cells/μL (150/175 × 10^6^/L) at two consecutive time points during the first year of transplantation, which categorized only about 10% of included patients as having high TCR γδ cell reconstitution but in a wide time frame. In our study, clinical impact was shown by day 56 by a dichotomization of the median concentration values of 21 × 10^6^/L preceding relapse in all patients and suggesting a protective effect of early robust TCR γδ cell immune reconstitution. TCR γδ cells were the only T cell subsets significantly associated with OS and RFS in after adjustment for transplant related factors and other cell subsets, 56 days after transplantation. The overall day 56 CD3 cell concentration was not significantly associated with clinical outcomes, and thus the association of increasing concentrations of TCR γδ T cells suggest a “dose-response” relationship to the absolute concentration of this subset particularly. Day 56 TCR γδ cell concentrations were furthermore the only T cell subsets significantly associated with death from relapse. However, in sensitivity analysis, including cell subsets as time-updated variables, all T cell subsets together with the overall CD3 concentrations were significantly associated with both OS and RFS. This reflects how a more long-term robust T cell concentration in general is important for outcomes after transplantation. The specific effect of TCR γδ cells early after transplantation may reflect the fact that, in opposition to CD4 and CD8 cells, this innate-like T cell type reconstitutes early and efficiently post-transplant (24, 25) and therefore might be protective of early relapse. Further studies are necessary to support this hypothesis.

Statistical associations for the TCR Vδ2 subtype closely followed those of the overall TCR γδ cell concentrations. This was not surprising as TCR Vδ2 constitutes the majority of TCR γδ cells in peripheral blood and is logically an important study subject for anti-tumor immunotherapy (26). Although low in fractions and concentrations, the TCR Vδ1 subset which also has reported graft-vs. leukemia effects (27, 28), was also significantly associated with RFS and OS by dichotomization of the day 56 median value in our study.

**Figure 8:**
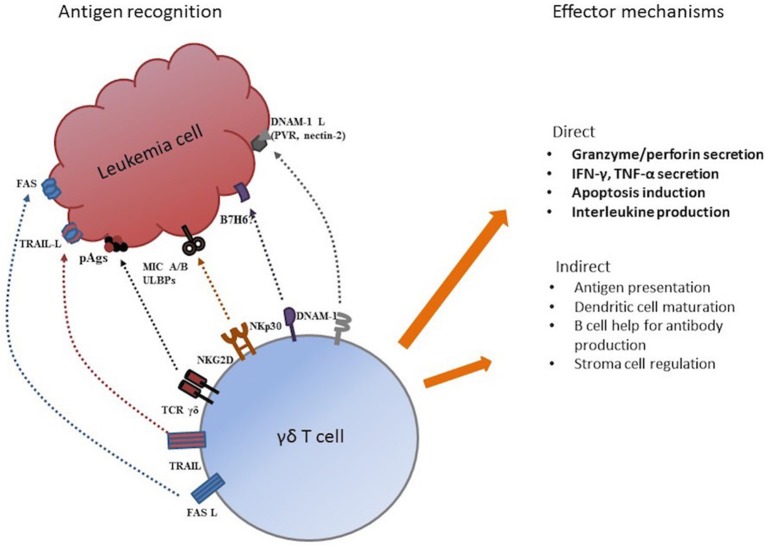
Possible mechanism for tumor antigen recognition and effector functions by TCR γδ cells. Adapted from Minculescu and Sengeloev (10).

Several possible mechanisms may be involved in tumor recognition and elimination by TCR γδ cells. Through their HLA-independent TCR γδ receptor, TCR γδ cells recognize small, non-peptide, phosphorylated antigens, “phospo-antigens” (pAgs), that are metabolites of metabolic pathways of practically all living organisms including humans (5, 29). Hypothetically, pAg-recognition allows discrimination between resting and malignant transformed cells facilitating TCR γδ cell tumor recognition (30). Other possible mechanisms for tumor recognition are through receptors from the natural cytotoxicity (NCR) family like NKp30 (31), the DNAM1/DNAM-1 receptor-ligand recognition (32, 33), and receptors from the NK receptor family like the mentioned NKG2D-receptor that recognized “stressed” or malignant transformed cells by activation through MHC-class I-related molecules MIC-A and MIC-B (6, 34) and UL16-binding proteins (ULBPs) (35, 36). As expected, the vast majority of our patient population (>99%) had TCR γδ cells expressing NKG2D. Effector functions of TCR γδ cell include direct cytotoxicity through perforin/granzyme secretion, death receptor ligands (Fas-L, tumor necrosis factor-related apoptosis-inducing ligand, TRAIL) and cytokine and interleukin production (4). Indirect effector mechanism involve antigen presentation (37, 38), regulation of the tumor microenvironment (39) and interactions with dendritic and stromal cells (4, 5). Figure 8 shows possible mechanisms for tumor recognition and effector mechanisms mediated by TCR γδ cells.

We observed a significantly lower risk of aGVHD in patients with high concentrations of TCR γδ cells 28 days after transplantation. Other studies have investigated potential effects of TCR γδ cell immune reconstitution on GVHD (12, 13, 19). Our study is, to our knowledge, the first to demonstrate clinical results suggesting a protective effect of early high TCR γδ cell concentrations, especially by the effector memory subset. Possible immunoregulatory properties of TCR γδ cells, together with the observation that high *fractions* of the TCR γδ cells of the total CD3 T cell compartment was associated with a lower less risk of aGVHD, suggest a protective effect of a high ratio of “innate/alloreactive” T cells. Further studies are needed to uncover the possible cellular mechanisms involved and potentially tolerogenic properties of TCR γδ cells.

While the immune reconstitution of TCR γδ cells did not impact the incidence of cGVHD, we found a significantly association with high CD4 T cell concentrations at day 56 and cGVHD. The analyses of differentiation subsets showed, that is was high concentration of naïve CD4 T cells, which correlated to increased cGVHD. We found no association to CD8 T cells, including the naïve subset; naïve T cells have, however, earlier been associated with increased risk of chronic GVHD (40).

A functional characterization of subsets and differentiation of TCR γδ cells was provided in this study. Analysis of differentiation subsets during immune reconstitution showed how the TCR γδ cell compartment consisted primarily of effector memory and TEMRA cells throughout the first year after transplantation. In this context, the TCR γδ cell population resembled the CD8 T cell population, the latter however, having substantially higher percentages of naïve cells especially early after transplantation. The CD4 compartment exhibited a relatively stable and high percentage of central memory cells which were not found in the CD8 or TCR γδ cell compartment. It was previously described, how peripheral expansion of effector memory cells in the CD8 compartment exceeds that of the CD4 compartment, presumably due to the CD8 reaction to herpes and other viruses which is most likely also the reason for the inversion of the CD4/CD8 ratio during the first year post-transplant (41). In the TCR γδ cell compartment, TEMRA cells expanded up to 6 months after transplantation, at which point this subset actually comprised more than half of the total TCR γδ cell compartment. One year after transplantation the TEMRA and effector memory subsets were again equally large as early after HSCT. We found CMV infection after transplantation to be associated with a trend toward higher percentages of TEMRA cells 6 months after transplantation which could be part of the reason for the expanding TEMRA subset. The expansion of the TEMRA subset was primarily at the expense of significantly lower percentages of the central memory subset, however this constitutes a minority of the total population in all patients (<5%) at all analyzed time points.

CMV infection was significantly associated with both higher absolute concentration and percentages of the Vδ1 subset 6 and 12 months after transplantation; the fraction of nonVδ1-nonVδ2 subset/TCR γδ cells was also significantly higher in CMV-infected recipients 12 months after transplantation. This supports the conception of a role of nonVδ2 cells in CMV infections as earlier described (25, 42). In 2011 the somewhat paradoxical association between CMV replication after transplantation and decreased relapse risk in 266 AML patients was demonstrated (43). Two years later, the study by Scheper et al. (44) demonstrating how TCR γδ cells are capable of cross-recognizing CMV and leukemia cells lead to the suggestion that Vδ2 negative cells contributed to the link between CMV reactivation and decreased relapse risk. A recent study demonstrated lower incidence of relapse following early CMV reactivation in myeloproliferative disorders (45). However, as the 2016 CIBMTR study (46), we could not demonstrate an impact of CMV on relapse-free survival, which we additionally analyzed in MDS and AML patients separately due to results from the previous study. However, data on CMV viral load or qualitative replication was not accessible in this study which also comprised a more heterogenous population, with only 66 patients when only myeloproliferative diseases were included. While post-transplant CMV infection affected TCR γδ cell immune reconstitution 6 and 12 months after HSCT, positive serological CMV status of the donor was highly associated with increased absolute concentrations and percentages of Vδ1 and nonVδ1-nonVδ2 cells the first 3 months post-transplant. Whether the latter in facts represents the original CMV status of the recipient before transplantation (as two thirds of all patients where serologically matched with their donors) or early donor reconstitution of the immune system, is not clear as chimerism analyses were not included in our study—and to our knowledge have never been performed on TCR γδ cells. Several other infections might have influenced the TCR γδ cells immune reconstitution, though due to patient numbers, other infections than CMV were not analyzed in this study.

Reported results of the clinical impact of TCR γδ cell reconstitution often originate from T cell depleted (pan- or TCR αβ) transplant settings (12, 14). The T cell replete setting of our study suggest an independent effect in the presence of conventional αβ T cells early after transplantation.

Recent studies focus on detailed immune reconstitution and optimal manufacturing procedures in order to design and implement post-transplant TCR γδ immune-therapy (25, 47, 48). Marcu-Malina et al. (49) have established ways of optimizing the response of TCR αβ T cells against cancer cells by transfer of tumor-reactive TCR γδ-receptors, and a recently published study presents the development of chimeric-antigen-receptor (CAR)—engineered TCR γδ cells (50). Our results support the rationale for further developing post-transplant immune-therapy exploiting innate and anti-tumor effects of TCR γδ T cells.

The shared innate characteristics and effector mechanisms of TCR γδ cells and NK cells categorize both cell types as possible anti-tumorous effector cells in HSCT (51–53), and both cell types reconstitute early after HSCT (24, 54). The earlier described high concentrations of the CD56bright subset early after transplantation (54) was also observed in this study, and high early concentrations of this subset in particular was significantly associated with improved OS and RFS with less risk of death from relapse after transplantation. The CD56bright subset comprises an immature NK cell subset with high capacity for proliferation but low natural cytotoxicity compared to CD56dim NK cell populations (55). After HSCT, however, the CD56bright NK cell subset population represent an increased fraction of the total NK cell compartment with reported overexpression of activating receptors and increased production of IFN-γ compared to donors (56) in accordance with a possible GVT-effect preventing relapse. Conflicting results have been reported on the role of NK cells in GVHD (57), but our results support the view of a regulatory role, as high CD56bright NK cells were significantly associated with less risk of aGVHD in our patient population. In the analyses of cell subsets included as time-updated variables, the overall NK cell concentration and the CD16/56 subsets were significantly associated with outcomes, perhaps in accordance with a gradual development and maturation of this dominating subtype after transplantation.

The strength of this study was the prospective design, standardized treatment protocols of a single-center institution, fixed sample time points, and available clinical data on all patients at ended follow-up time. The limitations of our study are the relatively heterogenous patient population in terms of disease. Also, although we present a functional characterization of TCR γδ cells, functional analyses of isolated TCR γδ cells demonstrating a specific graft-vs.-tumor effect on a cellular level have not been performed in this study. Clearly, this would support the proposed possibility of a causal relationship between TCR γδ cells immune reconstitution and relapse.

In conclusion, we report improved OS and RFS with less risk of relapse and aGVHD in patients with high concentrations of TCR γδ cells 2 months after transplantation, suggesting an active role of TCR γδ cells in the graft-vs.-tumor response after transplantation. Our clinical findings support further research of methods of improving the efficiency of post-transplant TCR γδ cell adoptive immune therapy in order to prevent relapse and improve survival after HSCT.

## Ethics Statement

The study was approved and carried out in accordance with the recommendation by the Danish National Committee on Health Research Ethics, and all participants gave written informed consent prior to transplantation in accordance with the Declaration of Helsinki.

## Author Contributions

LM performed the research, analyzed the data, and wrote the paper. HM and LR contributed with new reagent and analytic tools in the laboratory. NS, IS, LF, and EH performed the research. BK and SP performed the research, helped to write the paper, and analyzed the data. AF-N and HS designed the research. JR performed the statistical analyses and revised the manuscript as native-speaker. HS furthermore performed the research and analyzed the data.

### Conflict of Interest Statement

The authors declare that the research was conducted in the absence of any commercial or financial relationships that could be construed as a potential conflict of interest.
