# Supplementary material for: Improved Overall Survival, Relapse-Free-Survival, and Less Graft-vs.-Host-Disease in Patients With High Immune Reconstitution of TCR Gamma Delta Cells 2 Months After Allogeneic Stem Cell Transplantation
Source: Front Immunol. 2019 Aug 22;10:1997. doi: 10.3389/fimmu.2019.01997 (PMC6714591; doi:10.3389/fimmu.2019.01997)
Supplement: Supplementary file 1 [file Data_Sheet_1.docx]

**Supplemental data**

Table 1. Two-tube panel of monoclonal antibodies (mAB) used for leukocyte phenotyping. Antibodies were titrated and used in saturating concentrations.

| Flurochrome | mAB | Clone | Manufacturer  catalog no. |
| --- | --- | --- | --- |
| **Tube 1** |  |  |  |
| FITC | TCRαβ | Clone WT31 | BD-333140 |
| PE | TCRγδ | Clone 11F2 | BD-333141 |
| PerCp Cy5.5 | CD4 | Clone SK3 | BD-332772 |
| PE-Cy7 | CD45RA | Clone L48 | BD-337186 |
| Alexa Fluor 647 | CD197 | Clone 150503 | BD-560816 |
| - |  |  |  |
| APC-H7 | CD45RO | Clone UCHL1 | BD-561137 |
| V450 | HLA-DR | Clone L243 | BD-655874 |
| V500 | CD3 | Clone SP34-2 | BD-560770 |
| BV605 | CD8 | Clone SK1 | BD-564116 |
| **Tube 2** |  |  |  |
| FITC | TCRVδ2 | Clone 123R3 | Miltenyi-  130-095-798 |
| PE | TCRγδ | Clone 11F2 | BD-333141 |
| - |  |  |  |
| PE-Vio770 | TCRVδ1 | Clone REA173 | Miltenyi-  130-100-540 |
| APC | CD314 | Clone 1D11 | BD-558071 |
| - |  |  |  |
| APC-H7 | CD16 | Clone 3G8 | BD-560195 |
| V450 | CD56 | Clone B159 | BD-560360 |
| V500 | CD3 | Clone SP34-2 | BD-560770 |
| BV605 | CD337 | Clone p30-15 | BD-563384 |

Figure 1. Extracts from flow panels and gating strategies in tube 1 (page 3-4) and tube 2 (page 5-6). Dead cells/debris and dublets were remowed as shown in the dot plot forward and side scatter (FSC/SSC) and FSC Area/FSC High. Lymphocytes were identified based on their forward and side scatter properties. In tube 1, CD3pos events were selected on the CD3/SSC dot plot and subsequintly CD3 T cells were separated in a TCR αβ/TCR γδ plot and TCR αβ T cells were further separated in a CD4/CD8 plot (not shown). TCR αβ T cells, TCR γδ T cells and CD4- and CD8 T cells were separately investigated for differentation markers in a CD45RA/CD45RO plot for identification of CD45RAneg/CD45RApos memory cell phenotypes, and a CD45RA/CD197 plot for identification of central(CD45RAnegCD197pos)/effector(CD45RAneg/CD197neg) memory-, CD45RApos/CD197pos naive-, and CD45RApos/CD197neg TEMRA cell phenotypes. The expression of HLA-DR were investigated in histograms for TCR αβ T cells, TCR γδ T cells and CD4- and CD8 T cells separately. In tube 2, TCR γδ (CD3pos) T cells were identified in a CD3/TCR γδ plot, and TCR γδ T cells were furthermore separated in subtypes in a TCR Vδ2/TCR Vδ1 plot. NK cells were identified in a CD56/CD16 plot gated on CD3neg lymphocytes and 3 populations (CD56bright, CD16pos/CD56pos and CD16bright) were identified based on CD16- and CD56 expression. TCR γδ T cells, TCR Vδ1 T cells, TCR Vδ2 T cells, CD56bright NK cells, CD16pos/CD56pos NK cells and CD16bright NK cells were separately investigated for the expression of CD314 and CD337 in histograms and analyzed based on fractions of cells positive as well as the MFI (mean fluorescence intensity) expression.


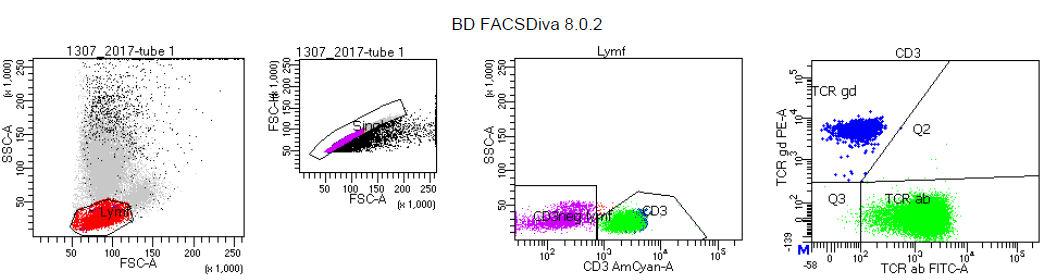


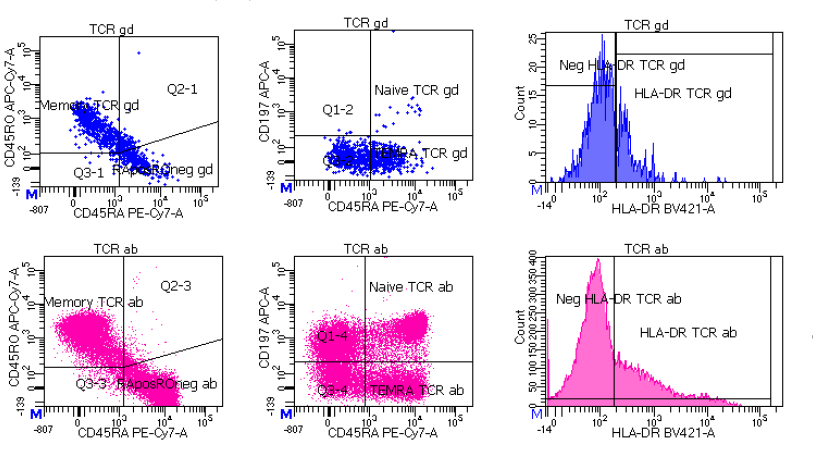


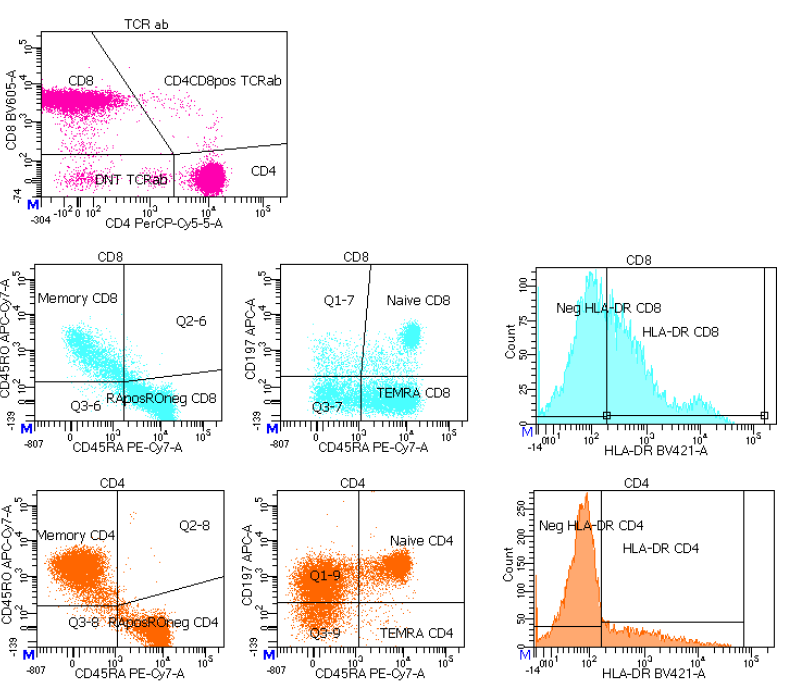


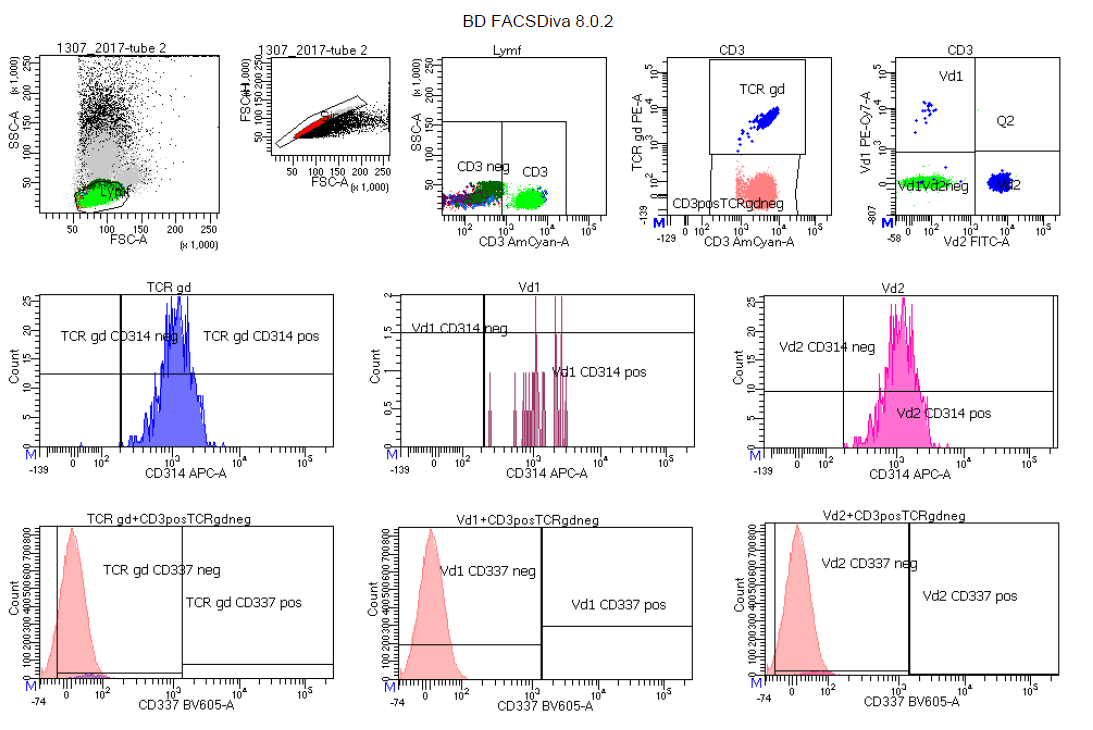


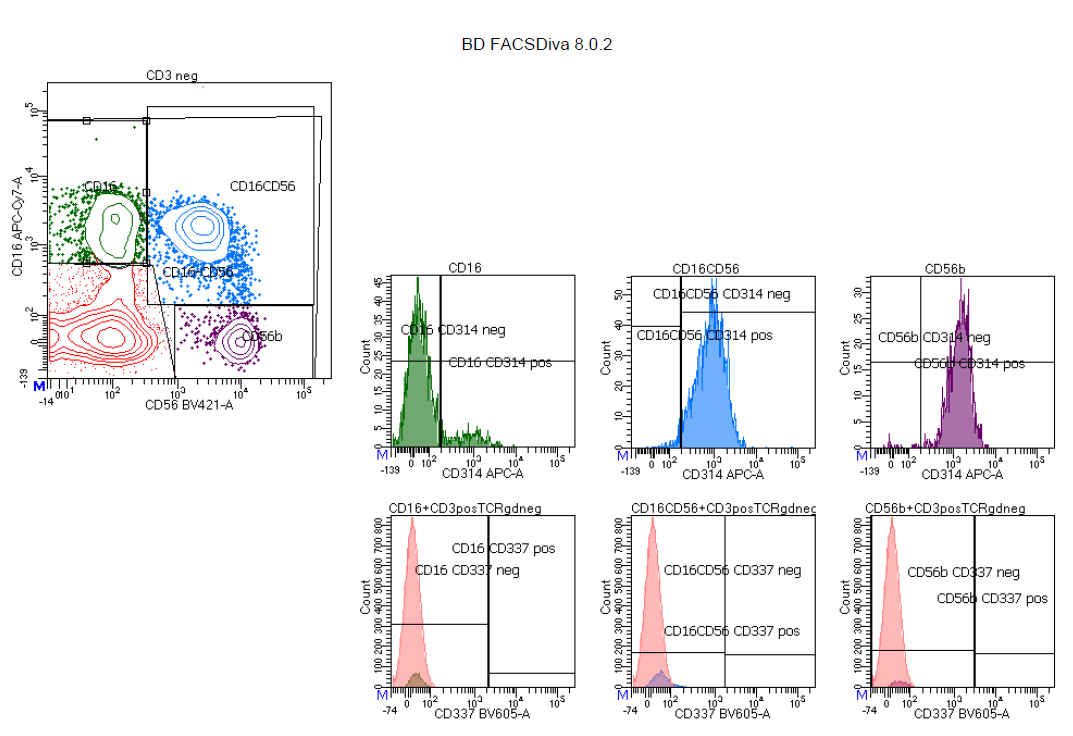


Table 2. Subset definitions and phenotypes. The absolute concentrations of CD3, CD4, CD8 and NK cells were calculated by the BD ^TM^ Trucount system. Additional fractions and concentrations were calculated from the panels in Table 1.

| Subset definition | Phenotype |
| --- | --- |
| CD3+TCR γδ+  CD3+TCR γδ+CD45RA+CD197+  CD3+TCR γδ+CD45RA-CD197+  CD3+TCR γδ+CD45RA-CD197-  CD3+TCR γδ+CD45RA+CD197-  CD3+TCR γδ+Vδ1+  CD3+TCR γδ Vδ2+  CD3+TCR γδ+Vδ1-Vδ2-  CD3+TCR αβ+CD4+  CD3+TCR αβ+ CD4+CD45RA+CD197+  CD3+TCR αβ+ CD4+CD45RA-CD197+  CD3+TCR αβ+ CD4+CD45RA-CD197-  CD3+TCR αβ+ CD4+CD45RA+CD197-  CD3+TCR αβ+CD8+  CD3+TCR αβ+ CD8+CD45RA+CD197+  CD3+TCR αβ+ CD8+CD45RA-CD197+  CD3+TCR αβ+ CD8+CD45RA-CD197-  CD3+TCR αβ+ CD8+CD45RA+CD197-  CD16/CD56+  CD16lowCD56++  CD16+CD56+  CD16++CD56low | TCR γδ cells  Naive TCR γδ cells  Central memory TCR γδ cells  Effector memory TCR γδ cells  TEMRA TCR γδ cells  TCR Vδ1 cells  TCR Vδ2 cells  TCR nonVδ1-nonVδ2  CD4 T cells  Naive CD4 T cells  Central memory CD4 T cells  Effector memory CD4 T cells  TEMRA CD4 T cells  CD8 T cells  Naive CD8 T cells  Central memory CD8 T cells  Effector memory CD8 T cells  TEMRA CD8 T cells  NK cells  CD56bright NK cells  CD16CD56 NK cells  CD16bright NK cells |
|  |  |
|  |  |
